# Supplementary material for: Microfabrication of a Chamber for High-Resolution, In Situ Imaging of the Whole Root for Plant–Microbe Interactions
Source: Int J Mol Sci. 2021 Jul 23;22(15):7880. doi: 10.3390/ijms22157880 (PMC8348081; doi:10.3390/ijms22157880)
Supplement: Supplementary file 1 [file ijms-22-07880-s001.zip › ijms-1302457-supplementary.pdf]

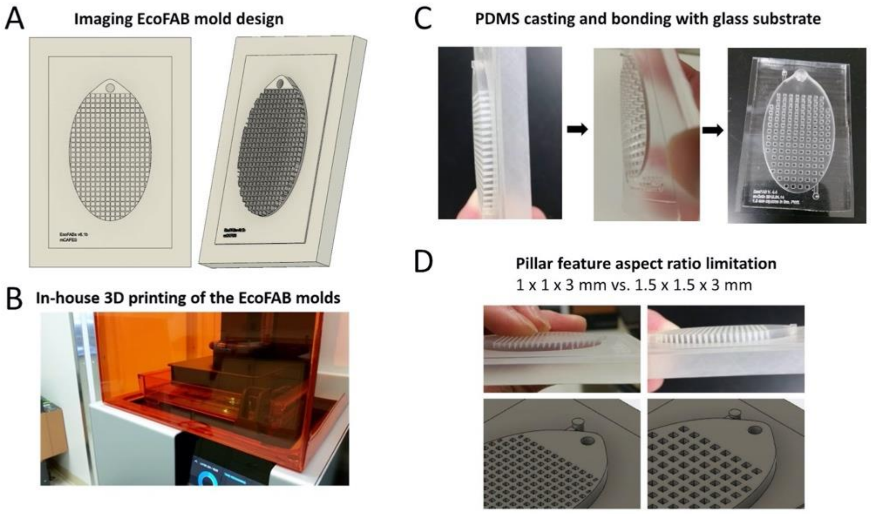

Figure S1. Imaging EcoFAB mold design and fabrication process. A) The rendered drawing of the Imaging EcoFAB mold design shown in two perspectives. B) The 3D printing of the EcoFAB mold by Form2 printer. C) PDMS casting of the mold and bonding with the glass substrate to complete the EcoFAB fabrication process. D) Demonstration of the printer's limitation regarding high aspect ratio features. In this case, the 1 x 1 x 3 mm pillar feature could not be printed as shown in the upper left inset while the 1.5 x 1.5 x 3 mm feature was adequately printed.

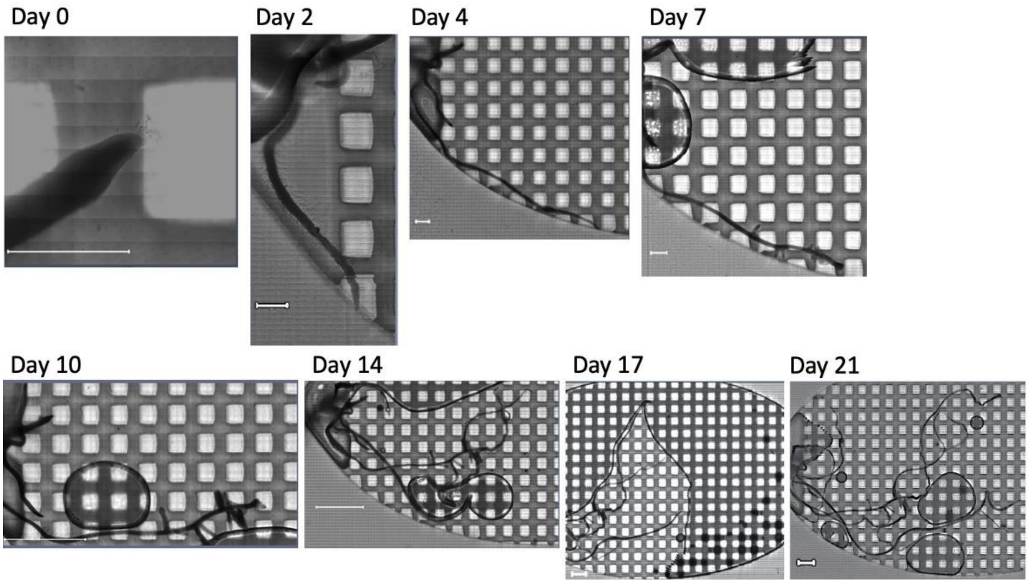

Figure S2. Time series images of B. distachyon in an Imaging EcoFAB. Images taken at 40x magnification. Time is recorded as days in the imaging EcoFAB. Seeds were germinated on a 1% agar water plate before transplanting to the imaging EcoFAB. The bars on days 0, 2, 4, and 7 are 1 mm. The bars on days 10, 14, 17, and 21 are 5 mm.

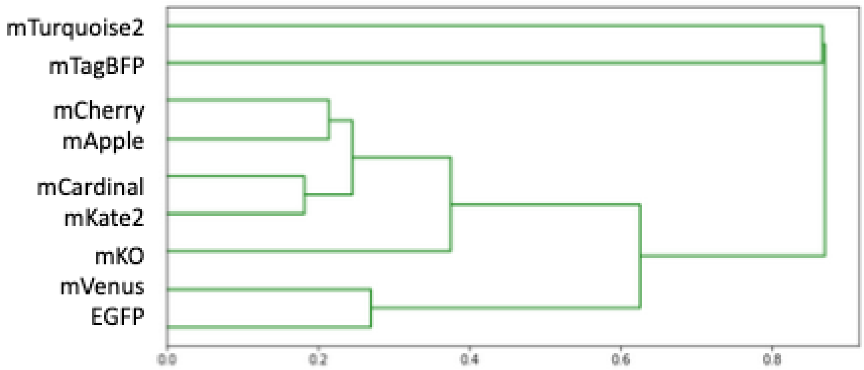

Figure S3. Grouping of fluorescent protein strains by K-means clustering from scikit-image package. The dendrogram shows which fluorescent proteins are distinguishable by computational analysis. Four bins resulted from this analysis: Red (mCherry, mApple, mCardinal, mKate2), Orange (mKO), Green (mVenus, EGFP), and background (mTAGBFP, mTurquoise2, background noise).

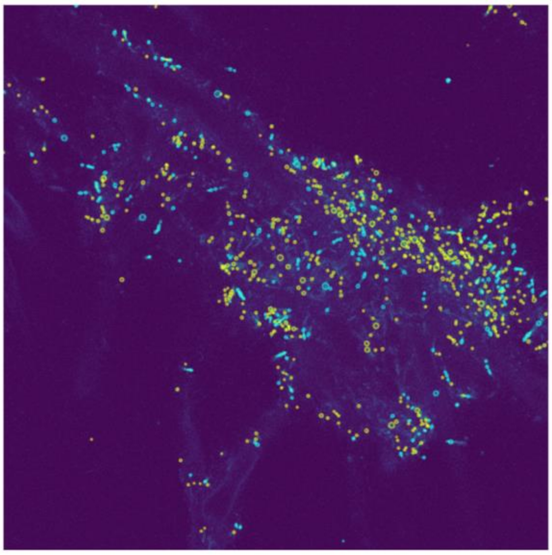

Figure S4. The segmentation analysis of the multispectral image of *P. simiae* (Figure 3C) from scikit-image package. The fluorescence signals were dilated and eroded using the circular structuring element to help distinguish the individual bacterium. The segmentation analysis counted a total of 478 cells in the image.

Analysis code: https://drive.google.com/drive/u/2/folders/1ohekfQyR2Rdwhyr-EyyU3dmVyMA586H3

Full image files: Image files

- Day00_40x.czi
- Day01_40x.czi
- Day02_40x.czi
- Day04_40x.czi
- Day07_40x.czi

- Day10_40x.czi
- Day14_40x.czi
- Day17_40x.czi
- Day21_40x.czi

Full root scans at 10x of the same plant/device as Figure 2

- Day00_10x.czi
- Day01_10x.czi
- Day02_10x.czi
- Day04_10x.czi
- Day07_10x.czi
- Day10_10x.czi
- Day14_10x.czi
- Day17_10x.czi
- Day21_10x.czi

Full root scans at 20x of the same plant/device as Figure 2

- Day00_20x.czi
- Day01_20x.czi
- Day02_20x.czi
- Day04_20x.czi
- Day07_20x.czi
- Day10_20x.czi
- Day14_20x.czi
- Day17_20x.czi
- Day21_20x.czi

Full images from Figure 3 (multispectral)

- Lambda40x.lsm
- Lambda20x.lsm
- Lambda10x.lsm

Full images from Figure 4 (succession)

- BFP05_40x.lsm

- BFP05mCherry01_40x.lsm

Full images from Figure 5 (media types)

- axenic_sand_10x.lsm
- h1rfp_phytagel_20x.lsm
